# Psychology as a Natural Science

**Published:** 1889-08

**Authors:** 


PSYCHOLOGY AS A NATURAL SCIENCE, applied to the solution of occult psychic phenomena. By C. G. Raue, M. D. Philadelphia, Porter & Coates, 1889.

The work embraces the consideration of the following important subjects:

Mind reading, thought transference, hypnotism, somnambulism, statuvolism, clairvoyance, second sight, retrospection, psychometery, telepathy, telergy, the double apparitions, phantasms of the living and dead, and spiritualistic phenomena, etc., discussed from an original standpoint.

Following Beneke, the great German investigator, Dr. Raue has endeavored to solve on the basis of the New Psychology, the occult psychic phenomena, claiming so much attention from modern thinkers. Dr. Raue proves that materialism is incompetent to explain these occult manifestations, and endeavors to give a rational psychological explanation of the same. The work is characterized by perfect fairness towards his opponents.

The author displays rare logical powers and a singularly happy way of making his foes convict themselves "out of their own mouths." Price, $3.50.

